# Trajectories of Concurrent Psychological Distress, Heavy Episodic Drinking and Daily Cigarette Smoking From Adolescence to Midlife: Patterns and Their Sociodemographic Correlates

**DOI:** 10.1177/14550725251408211

**Published:** 2026-01-02

**Authors:** Noora Berg, Maarit Piirtola, Mauri Marttunen, Antti Latvala, Olli Kiviruusu

**Affiliations:** 1 Department of Healthcare and Social Welfare, 3837 Finnish Institute for Health and Welfare, Helsinki, Finland; 2 Institute for Molecular Medicine Finland, HiLIFE, 3835 University of Helsinki, Helsinki, Finland; 3 UKK Institute for Health Promotion Research, Tampere, Finland; 4 Adolescent Psychiatry, Helsinki University and Helsinki University Hospital, Helsinki, Finland; 5 Institute of Criminology and Legal Policy, 3835 University of Helsinki, Helsinki, Finland

**Keywords:** alcohol use, heavy episodic drinking, life course, mental health, smoking

## Abstract

**Aim:**

The present study examined the joint development and co-occurrence of psychological distress (mainly depressive and anxiety symptoms), heavy episodic drinking (HED) and daily cigarette smoking from adolescence to adulthood.

**Methods:**

Data were drawn from two prospective longitudinal cohorts: the TAM study (*N* = 2194; ages 16–52 years) and the FinnTwin16 study (*N* = 5563; ages 16–35 years). Latent class analysis identified distinct trajectory groups, and multinomial regression was used to examine associations with sociodemographic factors.

**Results:**

Across both cohorts, several trajectory groups emerged: low levels of all three health concerns, high levels of all and high distress with low-to-moderate substance use. In the TAM cohort with longer follow-up time, additional groups included increasing HED and a group indicating moderate levels of all with a peak in daily cigarette smoking. Sociodemographic factors were associated with trajectory group membership. Men were more likely to belong to groups characterized by higher HED and lower distress, while women were more often in groups with higher distress and lower substance use. Participants from non-nuclear families were more likely to belong to groups with elevated substance use or distress. Low parental socioeconomic position was associated with increased likelihood of belonging to high-risk groups in both cohorts.

**Conclusions:**

These findings highlight the importance of considering the interplay between substance use and mental health across the life course, as well as the role of early sociodemographic context in shaping these trajectories. Tailored interventions and treatment should account for these differing developmental patterns and background factors.

## Introduction

Poor mental health and high levels of alcohol and tobacco consumption are major public health concerns (Peacock et al., 2018; Vigo et al., 2016). Heavy alcohol use and cigarette smoking are more prevalent among individuals experiencing psychological distress (Boden & Fergusson 2011; Weinberger et al., 2017). Although the mechanisms underlying this association, including the direction of possible causal effects, are not fully understood, both substance use and poor mental health have been identified as risk factors for each other (Boden & Fergusson, 2011; Copeland et al., 2013; Esmaeelzadeh et al., 2018; Kushner et al., 2000; Mathew et al., 2017; Swendsen & Merikangas 2000). High levels of alcohol and tobacco use also contribute to lower life expectancy among individuals with mental disorders (Wahlbeck et al. 2011). To address this cumulative burden, new perspectives are needed on this well-acknowledged association between mental health and substance use. Life course theory offers an interdisciplinary framework that integrates individual, contextual and temporal dimensions to better understand the co-development of psychological distress and heavy substance use (Shanahan et al., 2016). The review of prior literature is structured around conceptual and empirical themes outlined by Schulenberg et al. (2018) who emphasize a developmental perspective on substance use. Key themes include: (1) age-related patterns (age curves); (2) heterogeneity in the developmental trajectories; (3) embeddedness within broader developmental processes; and (4) predictors of change.

### Age Curves of Alcohol Use, Daily Cigarette Smoking and Psychological Distress

Research increasingly focuses on stability and change in psychological distress/symptoms/disorders and substance use across time. Typically, substance use increases during adolescence and young adulthood, then either decreases (alcohol) or stabilizes (tobacco) (Chassin et al., 2018; Schulenberg et al., 2018). Motivations for use also vary by age: experimentation is common in adolescence and young adulthood, while using substances to regulate stress and emotions becomes more prevalent in adulthood, increasing the risk of alcohol use disorders (Bakkali et al., 2023; Patrick et al., 2011). Mental health development varies by study design and focus (e.g., symptoms vs. diagnosis, prevalence vs. incidence, depressive symptoms vs. broader distress, cross-sectional vs. birth cohort vs. cohort sequential study design). Depression incidence tends to rise from childhood to adolescence, peaking in young adulthood (Pedersen et al., 2014). The prevalence follows a similar pattern in Western countries, though trends vary globally (Ferrari et al., 2013; Kessler & Bromet, 2013). For anxiety, the evidence is less clear. Generally, anxiety prevalence increases from adolescence to adulthood and then declines in older age, with variation across specific symptoms and disorders (Bandelow & Michaelis, 2015; Copeland et al., 2013; Costello et al., 2011; Martin, 2003; Wittchen et al., 1994). Genetic factors partly explain these age-related variations (Nivard et al. 2015). Regardless of measurement approach, both subclinical and clinical indicators of depression and anxiety are widespread and contribute significantly to global health burdens (Brody, 2018; GBD 2019 Mental Disorders Collaborators, 2022; Yang et al., 2021). In summary, both psychological distress and substance use tend to peak in young adulthood forming a shared age-related pattern.

### Heterogeneity in Age Curves (Trajectory Studies) and Their Mutual Associations

Studies have increasingly examined substance use and mental health trajectories and highlighted the heterogeneity in the age curve (Moulton et al., 2023; Paksarian et al., 2016; Schubert et al., 2017). However, few have examined how these trajectories are embedded within broader developmental processes, particularly the joint development of psychological distress, alcohol use and cigarette smoking. Simultaneous modeling of multiple related outcomes offers a nuanced yet interpretable summary of their developmental interconnections (Nagin & Odgers, 2010). Understanding heterogeneity in joint trajectories is important for identifying potential subgroups with distinct, age-specific needs for prevention and treatment. For example, Lee et al. (2018) examined joint trajectories of alcohol use, tobacco use and depressive symptoms from adolescence to adulthood (ages 14–29 years) among African Americans and Puerto Ricans born in the mid-1970s in the Harlem Longitudinal Development Study. They identified five trajectory groups: (1) moderate alcohol use, high tobacco use and high depressive symptoms (12%); (2) moderate alcohol use, high tobacco use and low depressive symptoms (26%); (3) moderate alcohol use, low tobacco use and low depressive symptoms (18%); (4) low alcohol use, no tobacco use and high depressive symptoms (11%); and (5) low alcohol use, no tobacco use and low depressive symptoms (33%).

Although studies modeling all three phenomena are rare, evidence from dual-trajectory studies supports their intertwined development. An Australian age-period-cohort study concluded that heavy episodic drinking (HED) (≥4 drinks in a row) and psychological distress remained positively associated across the lifespan (Halladay et al. 2023). Our previous Finnish study examined whether the development of psychological symptoms differed between persons with different developmental paths of HED from adolescence to midlife and revealed that the greater the HED trajectory indicated frequent HED, the higher was the level of symptoms throughout the follow-up (Berg et al., 2019).

In another study with 30 years of follow-up, persistent daily smoking from young adulthood to midlife was associated with elevated depressive symptoms, particularly among men (Kiviruusu et al., 2024). Similarly, another longitudinal study with a 10-year follow-up found that lifetime stress and anxiety disorders were more strongly associated with smoking trajectories than to non-smoking trajectory (Goodwin et al., 2013).

Alcohol and tobacco use are associated, and they are intertwined at physiological, psychological and social levels (Bien & Burge, 1990; Room, 2004). Many studies have found positive correlations between their joint trajectories, although most follow-ups end in young adulthood, at the peak period of substance use (Cance et al., 2017; Jackson et al., 2000, 2009; Schweizer et al., 2014).

### Sociodemographic Characteristics

Understanding individual characteristics (e.g., sex) and early-life background factors (e.g., socioeconomic position (SEP)) is essential for identifying developmental pathways of mental health and substance use. This knowledge helps to better target interventions aimed at preventing comorbidity and the persistence of these problems over time.

#### Sex

Men generally use alcohol and tobacco in larger amounts and more often than women, while women tend to report higher levels of psychological distress (Kuehner, 2017; Zahn-Waxler et al., 2008). These sex differences may reflect gendered responses to negative affect (Martin et al., 2013). Internalizing symptoms linked to emotion regulation difficulties are more typical in women, while externalizing symptoms associated with behavior control are more typical in men (Hussong et al., 2018; Kuehner, 2017). However, it remains unclear whether the longitudinal linkages between substance use and mental health differ by sex.

#### Socioeconomic Background

In a 20-year follow-up study, Lee et al. (2018) found that individuals in the trajectory group with low alcohol use, no tobacco use and low depressive symptoms had the highest educational level, while those in the group with moderate alcohol use, high tobacco use and high depressive symptoms had the lowest. More broadly, research has shown that (1) individuals with higher SEP may consume similar or greater amounts of alcohol than those with lower SEP, but the latter group experiences more negative consequences (Collins, 2016); (2) tobacco use is more prevalent among lower SEP groups (Hiscock et al., 2012); and (3) socioeconomically disadvantaged individuals are more likely to develop mental health problems, although the types of problems may vary (Reiss, 2013).

### Aim

The present study addresses gaps in previous research, which often lack follow-up beyond early adulthood and focuses on only one or two health outcomes. By examining the developmental course and co-occurrence of psychological distress, HED and daily cigarette smoking, we aim to provide a more comprehensive, multidimensional understanding of these interrelated phenomena (Xie et al., 2010). Co-occurring mental health and substance use problems are associated with a more severe course of illness and pose challenges for prevention and treatment strategies (Morisano et al., 2014). This developmental approach provides information that helps to design interventions for the prevention of comorbidity and to better tailor treatment services based on individual needs.

Using a life course perspective that integrates individual (e.g., psychological distress, substance use), contextual (e.g., family structure and SEP) and temporal (age and historical time (cohort differences)) dimensions, this study examines the joint development of psychological distress, daily cigarette smoking and HED from adolescence to midlife.

Research questions:

1. What types of trajectory groups of concurrent HED, daily cigarette smoking and psychological distress from adolescence to midlife can be identified?
2. How are sociodemographic factors (sex, adolescent family structure and parental socioeconomic background) associated with trajectory group membership?

Furthermore, the present study investigated whether similar combined trajectory groups could be identified in two distinct cohorts: the TAM cohort (born in the 1960s) (Berg et al., 2021) and the FinnTwin16 cohort (born in the 1970s) (Kaidesoja et al., 2019). Smoking initiation has historically been more common in the older cohorts, particularly among men (Helakorpi et al., 2004).

Based on prior research, we expected to find a rather large group with low levels of distress, HED and daily cigarette smoking, as well as a group with high levels of all three. Regarding other possible groups, we did not set specific hypotheses. We also anticipated that women would be more likely to be assigned to distress-oriented trajectory groups, and men and individuals with low SEP to substance use-oriented groups. Our study with two cohorts is a starting point for examining these questions with various longitudinal data. No specific hypothesis were made regarding cohort differences because this would require a larger number of cohorts. Previous research suggests that the health-related risks associated with twin status (e.g., prematurity, low birth weight) tend to diminish with age, allowing twin cohorts to be analyzed alongside other population-based designs (Christensen & McGue, 2022).

## Methods

### Study Population

This prospective multicohort study is based on two longitudinal datasets from Finland that have followed participants from adolescence into adulthood: the TAM cohort (*N* = 2194, 96.7% of the target population) (Berg et al., 2021) and the FinnTwin16 cohort (*N* = 5563, 86.5% of the target population) (Kaidesoja et al., 2019).

**Figure 1.**
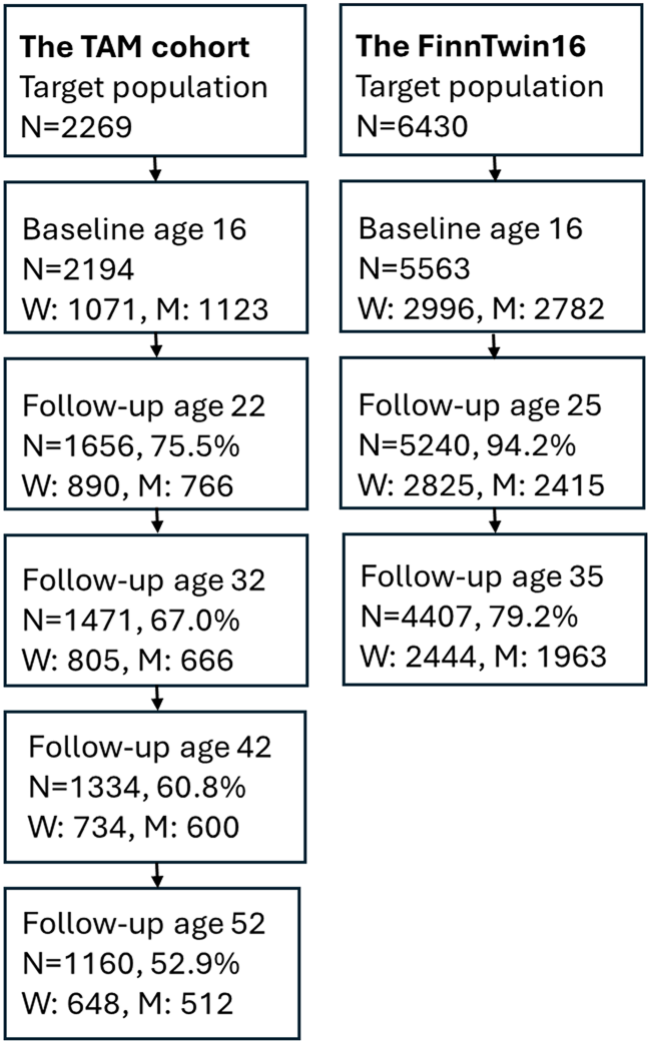
Flowchart of the Data Collections of the TAM Cohort and the FinnTwin16 Cohort.

The TAM cohort consists of Finnish-speaking pupils who attended the final year of compulsory school in 1983 in Tampere, a city in southern Finland. The participants completed the baseline questionnaire at age 16 years in school settings and were followed up via postal questionnaires at ages 22, 32, 42 and 52 years (in 1989, 1999, 2009 and 2019). FinnTwin16 is a population-based twin study comprising five consecutive birth cohorts (1975–1979). The target population was ascertained from the Central Population Register of Finland and was contacted via mail. Participants completed questionnaires at ages 16, 25 and 35 years (in 1991–1995, 2000–2002 and 2010–2012) (Figure 1). Participation rates were generally high: in the TAM cohort, 53.0% of the original participants responded at age 52 years; in the FinnTwin16 cohort, 72.0% of those contacted participated at age 35 years.

The TAM study was approved by the Institutional review board of the Finnish Institute for Health and Welfare. FinnTwin16 was approved by the Ethical Committee of the Department of Public Health, University of Helsinki, Helsinki University Central Hospital ethical committee, and by the Institutional Review Board of Indiana University. All participants provided their informed consent to participate.

### Measures

#### Psychological Distress

Psychological distress was operationalized primarily through symptoms of depression and anxiety. In the TAM cohort, a continuous measure was derived from a 17-item psychosomatic symptoms checklist, using seven items (rated 0−3) indicative of depression and anxiety (lack of energy, sleeping difficulties, nightmares, fatigue, irritability, loss of appetite and nervousness/anxiety). This measure has been used earlier and described in more detail by Pelkonen et al. (2003). Cronbach's alpha values showed acceptable/good coefficients ranging from 0.73 to 0.81 across waves. In the FinnTwin16 cohort, psychological distress at age 16 years was assessed using a four-item symptom list (tension or nervousness, fatigue, irritability, sleeping disorders and Cronbach's alpha 0.66. At ages 25 and 35 years, the validated General Health Questionnaire (i.e., GHQ-12) (Goldberg, 1972) was used. Although reliability at age 16 years is not optimal, it was considered acceptable given the priority placed on harmonizing measures across cohorts. Higher scores indicated greater distress. All symptom measures were standardized for analysis.

#### HED

HED was classified as (1) consuming more than five or six drinks on a single occasion or being drunk monthly or more often and (2) drinking the amount/being drunk less frequently or not at all. In the TAM cohort, HED was measured as the frequency of intoxication (16 and 22 years) and having six or more drinks in a row (32, 42 and 52 years). At age 16 years, the HED category included those who reported being drunk at least four times during the school term (on average once a month). At age 22 years, the HED category included those who reported heavy drunkenness at least once a month. At ages 32, 42 and 52 years, HED was based on the third item of the Alcohol Use Disorders Identification Test (AUDIT) (Babor et al., 2001); those who reported having six or more drinks in a session at least once a month were classified as heavy drinkers.

In the FinnTwin16 study, HED was measured as the frequency of intoxication (16 years) and having five or more drinks in a row (25 and 35 years). At age 16 years, the HED category included those who reported heavy drunkenness at least once a month. At ages 25 and 35 years, the HED category included those who reported having five or more bottles of beer, more than a bottle of wine or more than half a bottle of hard liquor (or a corresponding amount of alcohol) monthly or more often.

#### Daily Cigarette Smoking

Smoking status was classified as daily cigarette smoking vs. other smoking statuses (including never smoked, occasional smoking and former smokers).

#### Sociodemographic Predictors

Sociodemographic variables included sex, family structure and parental SEP at age 16 years. Family structure was categorized into (1) living with both mother and father; (2) living with a parent and a stepparent; and (3) other. Parental SEP was based on parental occupation (in TAM participants’ self-reports and in FinnTwin16 parents’ self-reports). SEP was classified into low (manual work), intermediate (lower non-manual work) and high (upper non-manual work) occupational positions. If the occupation of neither parent was available, parental education was used and similarly categorized as low (no vocational education), intermediate, or high.

### Statistical Analysis

Joint trajectories of HED, daily cigarette smoking, and psychological distress were identified using latent class analysis (LCA) in Mplus version 8.7 (https://www.statmodel.com). Participants were grouped based on their longitudinal profiles of these three variables at ages 16, 22, 32, 42 and 52 years in the TAM cohort and at ages 16, 25 and 35 years in the FinnTwin16 cohort. The models with 1–8 latent classes were estimated. The Akaike information criterion (AIC), Bayesian information criterion (BIC), sample size adjusted BIC (ssaBIC) and Lo–Mendell–Rubin likelihood ratio test (LMR) were used as the statistical criteria to determine the optimal number of classes. Emphasis was also placed on group size (> 5%) and interpretation. Participants were assigned to the latent classes according to the most likely group membership based on estimated posterior probabilities.

For the LCA, we included participants who had responded to questions about HED, daily cigarette smoking and distress in at least two study waves (TAM, *N* = 1943; FinnTwin16, *N* = 5166). To deal with missing values due to attrition, we used the full information maximum likelihood estimation method. The nature of the twin data (i.e., within-pair/family-level clustering) was accounted for in LCA using the Mplus cluster option.

To examine associations between sociodemographic predictors and trajectory group membership, we conducted multinomial regression analysis. In FinnTwin16, family-level clustering was addressed using a generalized linear mixture model framework.

Two sets of sensitivity analyses were performed. First, to account for differences in follow-up duration between cohorts, we repeated the analyses using harmonized time-points (ages 16, 22 and 32 years in TAM) to match the shorter follow-up in FinnTwin16. Second, trajectory analyses were also stratified by sex due to potential differences in symptom levels and substance use between women and men.

## Results

**Figure A1. fig4-14550725251408211:**
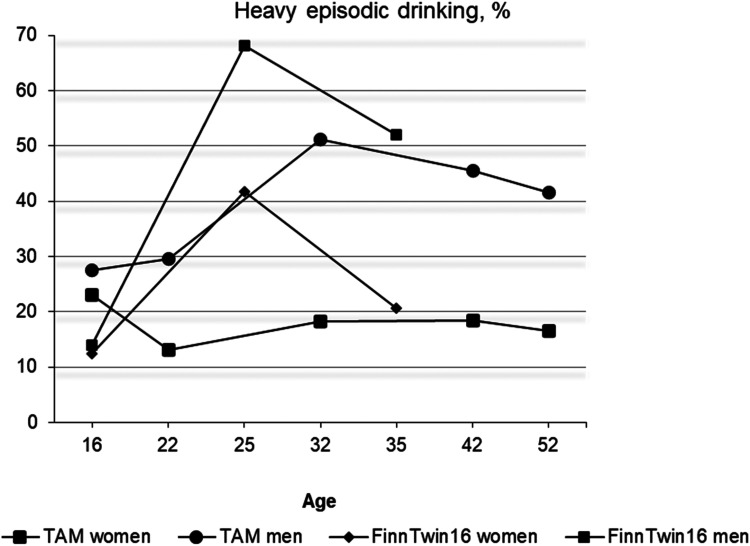
Prevalences of Heavy Episodic Drinking in Men and Women in the TAM and FinnTwin16 Cohorts.

**Figure A2. fig5-14550725251408211:**
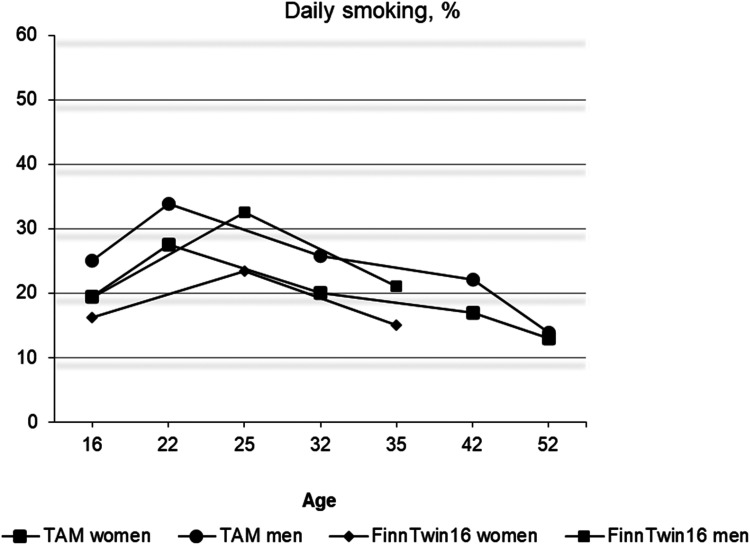
Prevalences of Daily Cigarette Smoking in Men and Women in the TAM and FinnTwin16 Cohorts.

**Figure A3. fig6-14550725251408211:**
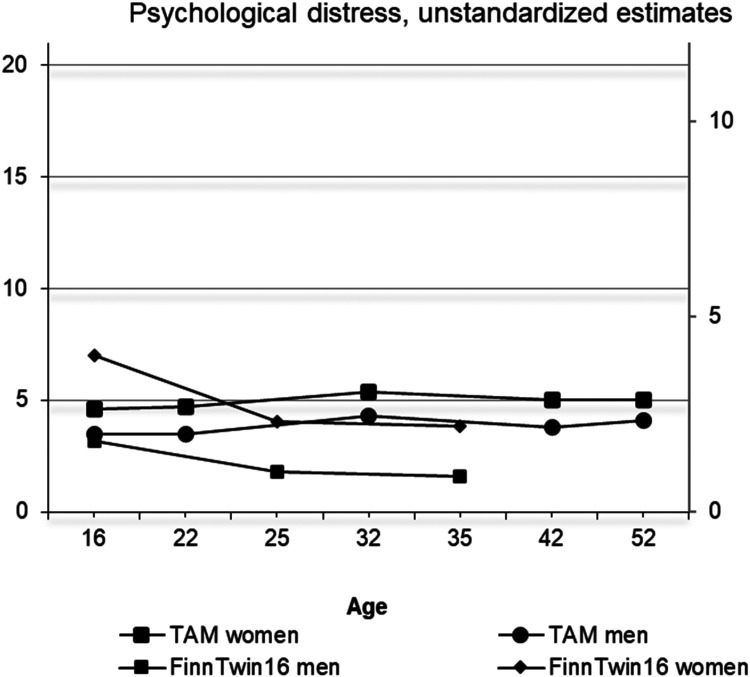
Unstandardized Means of Psychological Distress in Men and Women in the TAM and FinnTwin16 Cohorts. TAM Distress Scale (0–21) on the Left and FinnTwin16 scale (0–12) on the Right.

The descriptive statistics of the study variables are presented in Table 1 and the Appendix (Figures A1–A3). The means of psychological distress scales decreased from adolescence to adulthood in participants in the FinnTwin16 study and increased in the TAM study, peaking at age 32 years. The prevalence of HED in adolescence ranged from 12.4% to 23.0% among women and from 13.9% to 27.5% among men. In FinnTwin16, HED peaked at age 25 years and subsequently decreased, whereas in the TAM cohort, HED among women decreased at age 22 years, increased again at age 32 years and then stabilized. In the TAM cohort, HED among men increased until age 42 years. Daily cigarette smoking was most common in early adulthood (22/25 years, 23.5–33.9%) and decreased at later ages in both cohorts. Regarding sociodemographic background, participants in FinnTwin16 were less likely to come from low socioeconomic background (24%) compared to those in TAM (50%).

**Table 1.** Prevalence of Study Variables Among Women and Men in the TAM and FinnTwin16 Cohorts.

	TAM			FinnTwin16		
	Women,	Men,	Total,	Women,	Men,	Total,
Variables	Mean ± SD	Mean ± SD	Mean ± SD	Mean ± SD	Mean ± SD	Mean ± SD
Psychological distress age 16 years^a,b^	4.6 ± 2.7	3.5 ± 2.7	4.1 ± 2.8	4.0 ± 2.6	3.2 ± 2.5	3.6 ± 2.9
Psychological distress age 22/25 years^a,c^	4.7 ± 2.8	3.5 ± 2.9	4.1 ± 2.9	2.3 ± 2.9	1.8 ± 2.3	1.9 ± 2.7
Psychological distress age 32/35 years^a,c^	5.4 ± 2.9	4.3 ± 3.1	4.9 ± 3.1	2.2 ± 3.1	1.6 ± 2.7	2.0 ± 2.9
Psychological distress age 42 years^a^	5.0 ± 3.0	3.8 ± 3.1	4.5 ± 3.1			
Psychological distress age 52 years^a^	5.0 ± 3.2	4.1 ± 3.2	4.6 ± 3.3			
	% (*n*)	% (*n*)	% (*n*)	% (*n*)	% (*n*)	% (*n*)
HED^d^ age 16 years	23.0 (245)	27.5 (307)	25.3 (552)	12.4 (370)	13.9 (384)	13.1 (754)
HED age 22/25 years	13.1 (116)	29.6 (226)	20.8 (342)	41.7 (1174)	68.2 (1647)	53.9 (2821)
HED age 32/35 years	18.2 (146)	51.1 (339)	33.1 (485)	20.6 (499)	52 .0 (1008)	34.5 (1507)
HED age 42 years	18.5 (134)	45.6 (272)	30.7 (406)			
HED age 52 years	16.6 (107)	41.6 (212)	27.7 (319)			
Daily smoking age 16 years	19.4 (207)	25.1 (281)	22.3 (488)	16.3 (483)	19.3 (525)	17.7(1008)
Daily smoking age 22/25 years	27.5 (243)	33.9 (259)	30.5 (502)	23.5 (640)	32.5 (760)	27.7 (1400)
Daily smoking age 32/35 years	20.0 (161)	25.8 (171)	22.7 (332)	15.1 (353)	21.1 (392)	17.8 (745)
Daily smoking age 42 years	17.0 (125)	22.1 (132)	19.3 (257)			
Daily smoking age 52 years	13.0 (84)	13.9 (70)	13.4 (154)			
Parental socioeconomic position age 16 years						
Upper non-manual	17.9 (188)	19.5 (213)	18.7 (401)	35.3 (966)	33.9 (876)	34.6 (1842)
Lower non-manual	31.6 (332)	30.8 (336)	31.2 (668)	41.0 (1123)	41.0 (1059)	41.0 (2182)
Manual	50.5 (531)	49.7 (543)	50.1 (1074)	23.7 (650)	25.0 (646)	24.4 (1296)
Family structure age 16 years						
Mother + father	72.5 (738)	75.5 (794)	74.0 (1532)	80.4 (2397)	81.5 (2253)	89.9 (4650)
Parent + step parent	8.5 (87)	8.9 (94)	8.7 (181)	6.6 (196)	5.7 (158)	6.2 (354)
Other	19.0 (193)	15.6 (164)	17.2 (357)	13.1 (390)	12.8 (355)	13.0 (745)

a TAM:seven-item scale (range 0–21).

b FinnTwin16: four-item scale (range 0–12).

c FinnTwin16: GHQ-12 (range 0–12).

d Heavy episodic drinking.

**Figure 2.**
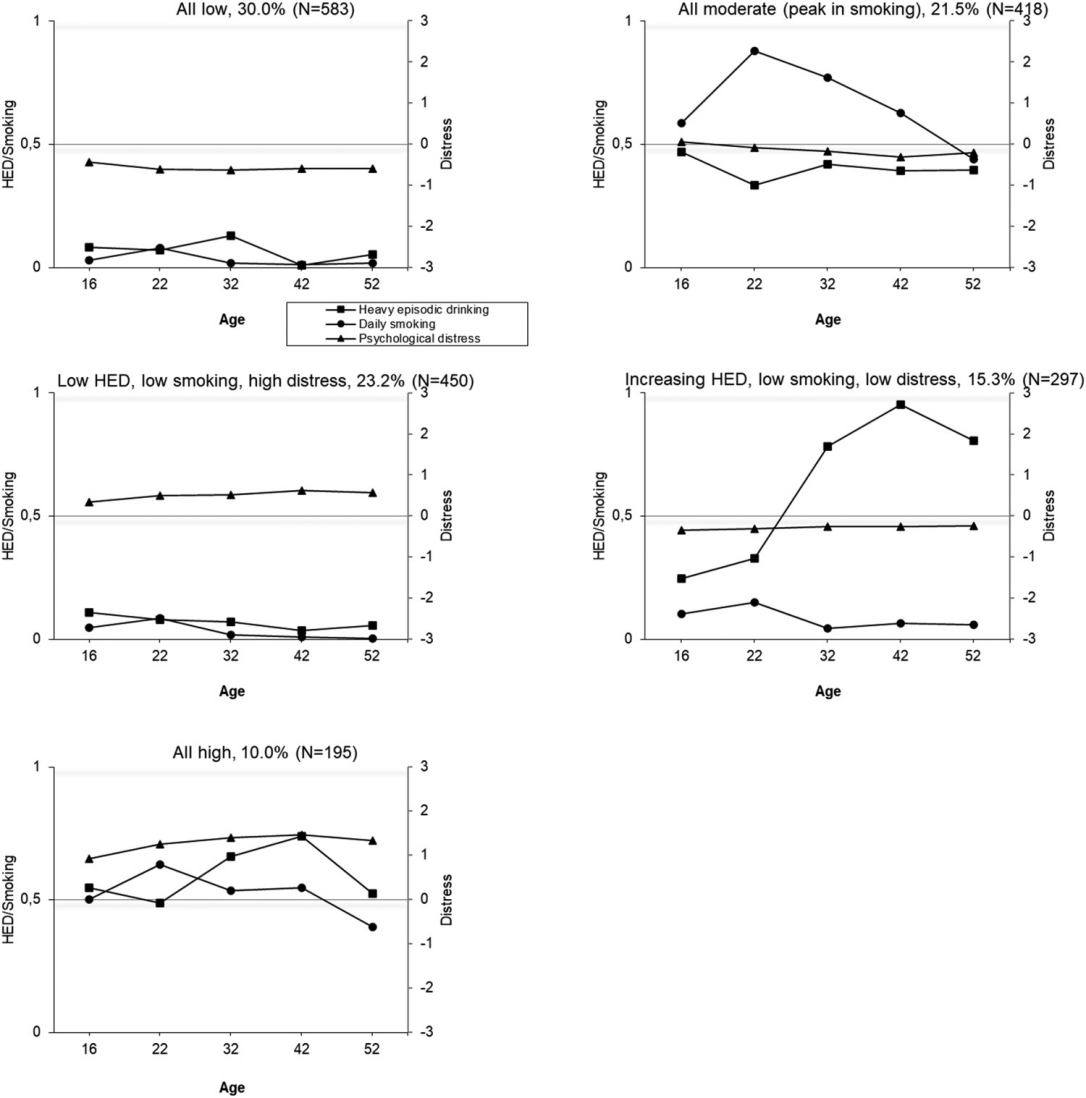
Conjoint Trajectories of Heavy Episodic Drinking (HED), Daily Cigarette Smoking and Psychological Distress Among the TAM Cohort at Ages 16-52 Years by (a) Class 1, (b) Class 2, (c) Class 3, etc. the Score for HED: 0 = Less than Monthly, 1 = Monthly or More Often; for Smoking: 0 = Less than Daily, 1 = Daily Smoking; for Psychological Distress: Standardized.

**Figure 3.**
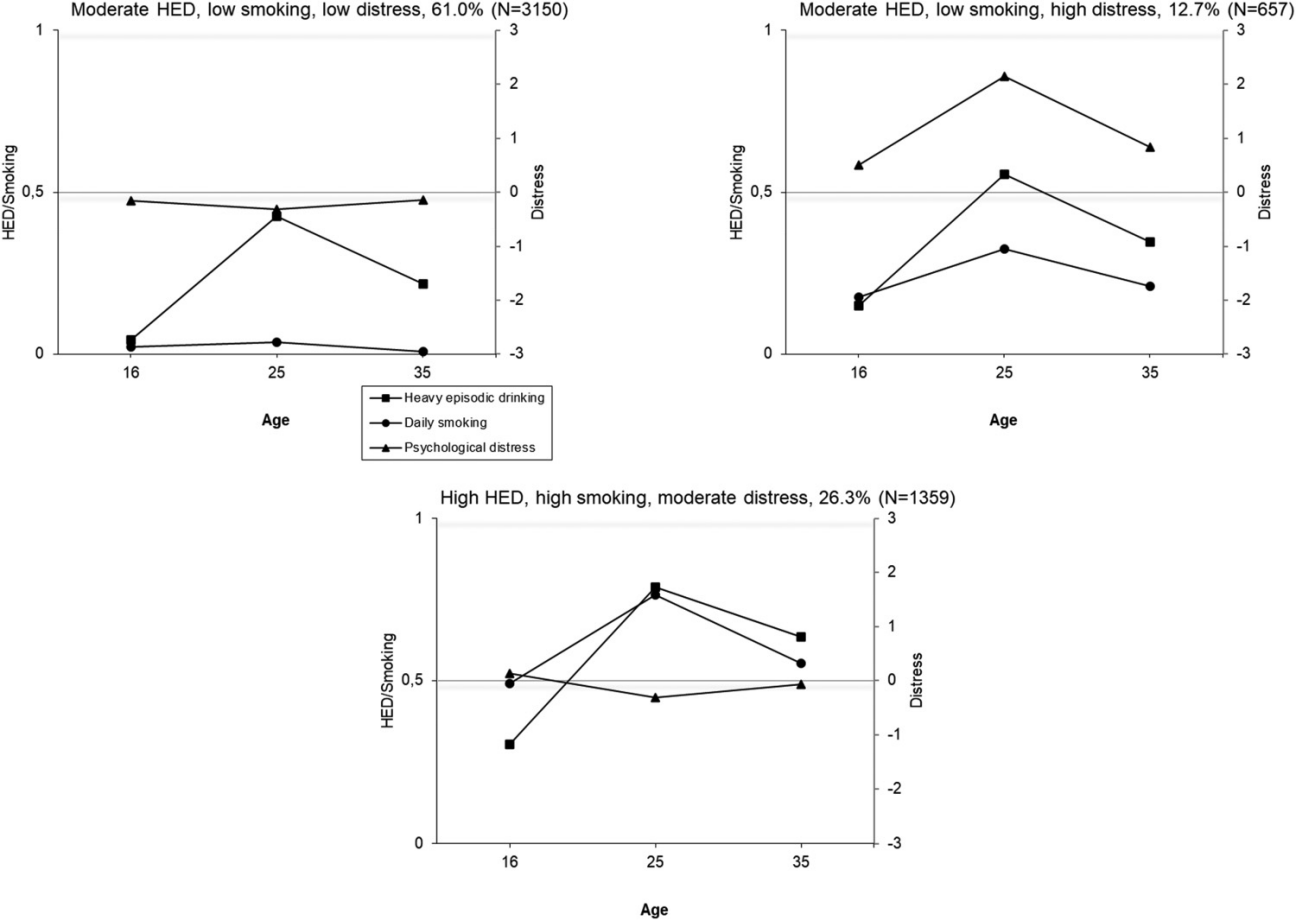
Conjoint Trajectories of Heavy Episodic Drinking (HED), Daily Cigarette Smoking and Psychological Distress Among the FinnTwin16 Cohort at Ages 16-35 Years by (a) Class 1, (b) Class 2, (c) Class 3, etc. The Score for HED: 0 = Less than Monthly, 1 = Monthly or More Often; for Smoking: 0 = Less than Daily, 1 = Daily Smoking; for Psychological Distress: Standardized.

**Figure A4. fig7-14550725251408211:**
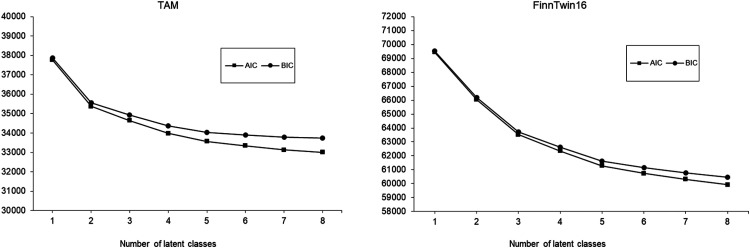
Fit Statistics (Akaike Information Criterion (AIC) and Bayesian Information Criterion (BIC)) by Number of Latent Classes and Cohorts.

Joint trajectories of HED, daily cigarette smoking and psychological distress were identified using latent class analyses. In both cohorts, the model fit indices (e.g., BIC), improved (decreased) as the number of classes increased, but larger numbers of classes resulted in very small group sizes (Table 2; see also the Appendix, Figure A4). A five-class solution in the TAM was selected because it showed good fit indices and reasonable group sizes (< 5%) (Table 2). For FinnTwin16, the optimal number of classes based on group size was a four-class model. However, a three-class model was chosen for subsequent analyses because it was interpretationally better in terms of producing groups with distinctively different profiles while maintaining large enough groups sizes (in the four-class solution, the “moderate HED, low daily smoking, high distress” group is split into two groups with similar longitudinal profiles, but some difference in the level; two other groups remain identical as in the three-class solution). The selected trajectory solutions are illustrated in Figures 2 and 3.

**Table 2.** Fit Statistics, Classification Indices and Class Sizes for Each Joint Trajectory Model of Heavy Episodic Drinking, Daily Cigarette Smoking and Psychological Distress.

*K*	Log Likelihood	AIC	BIC	SSABIC	LMR-LRT *p*	Entropy	Class Size (proportion based on model estimate)
*TAM*							
1	−18862.872	37765.743	37877.183	37813.643			100.0
2	−17649.088	35370.176	35570.767	35456.394	<0.001	0.810	65.1/34.9
3	−17272.262	34648.524	34938.268	34773.062	**0**.**0459**	0.798	59.7/26.6/13.8
4	−16926.256	33988.512	34367.407	34151.369	0.3236	0.755	25.0/38.2/11.1/25.7
5	−16698.522	33565.045	34033.092	33766.221	0.1532	0.755	**21.5/15.3/30.0/10.0/23.2**
6	−16574.474	33348.948	33906.147	33588.445	0.2710	0.772	17.6/30.2/23.4/13.7/12.9/2.2
7	−16450.706	33133.413	33779.763	33411.229	0.0739	0.780	10.9/29.5/6.1/10.6/18.1/22.8/2.1
8	−16371.797	**33007**.**593**	**33743**.**096**	**333.23.728**	–^a^	0.783	12.7/10.1/6.3/9.9/30.0/6.4/2.1/22.6
*FinnTwin16*							
1		69450.722	69529.320	69491.188			100
2	−32999.001	66042.002	66186.099	66116.190	<0.001	0.758	68.4/31.5
3	−31727.058	63518.116	63727.711	63626.026	<0.001	0.825	26.3/12.7/61.0
4	−31137.335	62358.669	62633.763	62500.301	<0.001	0.832	**56.7/10.8/24.7/7.7**
5	−30588.266	61280.532	61621.124	61455.886	<0.001	0.836	9.3/23.9/54.5/8.9/3.4
6	−30309.285	60742.570	61148.661	60951.646	0.0001	0.842	51.2/22.8/8.1/10.3/3.5/3.9
7	−30085–847	60315.693	60787.283	60558.491	<0.001	0.803	18.3/10.0/15.2/8.1/3.9/3.5/40.8
8	-29879.333	**59922**.**667**	**60459**.**755**	**60199**.**186**	**<0**.**001**	0.810	11.9/7.9/18.9/42.6/3.6/3.7/3.8/7.6

AIC = Akaike information criterion; BIC = Bayesian information criterion; *K* = number of classes; LL = log-likelihood; LMR-LRT = Lo–Mendell–Rubin likelihood ratio test; *p* = *p* value; SSABIC = sample size adjusted BIC. ^a^The likelihood ratio test could not be computed. The model did not converge.

In the TAM cohort, 53% of the participants were assigned to relatively stable trajectory groups (1) low levels of all three indicators; (2) low substance use and high distress; and (3) high levels of all three). The remaining two groups showed an increase in substance use (4) all moderate (peak in daily smoking) (22%) and (5) an increase in HED, low daily smoking and low distress (15%)). In the FinnTwin16, all trajectory groups showed at least a moderate peak at age 25 years in one or more indicators. The majority (61%) were assigned to a group indicating low levels of substance use and distress, with a peak in HED at age 25 years. The second largest group (26%) indicated high substance use, but low distress, and the third group (13%) indicated moderate or low substance use, but high distress.

The associations between sociodemographic variables and latent classes were analyzed using multinomial regression analysis (Table 3). In the TAM cohort, men were more likely assigned to groups with all moderate or increasing HED, low daily smoking and low distress. Women were more likely assigned to the group with low HED, low daily smoking and high distress. In FinnTwin16, men were more likely assigned to the high HED, high daily smoking and moderate distress group, and women to the moderate HED, low daily smoking and high distress group. Across both cohorts, participants from non-nuclear families were more often assigned to trajectory groups indicating high or moderate levels of substance use or distress compared to the group with low levels of use or distress. In the TAM, low parental SEP was associated with increased likelihood of belonging to groups of (1) all moderate; (2) low HED, low daily smoking and high distress; and (3) all high. In Finntwin16, low parental SEP was associated with being more likely assigned to the high HED, high daily smoking and moderate distress group.

**Table 3.** Sociodemographic Predictors of Joint Trajectories of Heavy Episodic Drinking, Daily Cigarette Smoking and Psychological Distress.^a^

	RRR (95% CI)	RRR (95% CI)	RRR (95% CI)	RRR (95% CI)
*TAM^b^*	All Moderate	Low HED, Low Daily Smoking, High Distress	Increasing HED, Low Daily Smoking, Low Distress	All High
				
Sex (man)	1.35 (1.05–1.74)	0.33 (0.25–0.43)	3.06 (2.25–4.16)	1.00 (0.72–1.38)
Family structure				
Mother + father	ref	ref	ref	ref
Parent + stepparent	4.51 (2.69–7.56)	2.04 (1.16–3.57)	2.32 (1.27–4.22)	6.73 (3.75–12.06)
Other	1.8 (1.27–2.56)	1.31 (0.92–1.86)	1.08 (0.71–1.63)	2.76 (1.81–4.22)
Parental SEP				
Upper non-manual	ref	ref	ref	ref
Lower non-manual	1.33 (0.91–1.96)	1.20 (0.80–1.75)	1.19 (0.80–1.75)	1.10 (0.67–1.81)
Manual	2.38 (1.67–3.40)	1.51 (1.09–2.10)	1.35 (0.93–1.95)	2.01 (1.28–3.15)
*FinnTwin16^c^*	Moderate HED, low Daily Smoking, High Distress	High HED, High Daily Smoking, Moderate Distress		
Sex (man)	.54 (.45-.65)	1.74 (1.51–2.01)		
Family structure				
Mother + father	ref	ref		
Parent + stepparent	1.41 (0.96–2.08)	2.53 (1.88–3.40)		
Other	1.75 (1.36–2.25)	1.84 (1.48–2.30)		
Parental SEP				
Upper non-manual	ref	ref		
Lower non-manual	0.87 (0.71–1.07)	1.34 (1.11–1.61)		
Manual	0.88 (0.71–1.07)	1.53 (1.24–1.89)		

CI = confidence interval; SEP = socioeconomic position.

a Relative risk ratios (RRRs) from multinomial logistic regression models (generalized linear mixture model for FinnTwin16). Bivariate models with only one predictor and trajectory group in the model at a time.

b Reference group All low.

c Reference group Moderate HED, low smoking, low distress.

## Discussion

The present study is among the first to examine the joint trajectories of HED, daily cigarette smoking and psychological distress across the life course, and to explore the nature of their co-occurrence. The findings demonstrate that, despite moderate to strong correlations between these outcomes, their joint development varies considerably. In both cohorts, distinct trajectory groups were identified: (1) low levels of all three health concerns; (2) high levels of all three; and (3) high distress with low-to-moderate substance use. In the older TAM cohort with longer follow-up time, two additional groups emerged: (4) increasing HED and a group indicating (5) moderate levels of all indicators, with a peak in daily smoking.

Schulenberg et al. (2018) propose a developmental framework for substance use within life course theory, which provides a useful perspective for interpreting these findings. One key component is the *age curve*, which reflects average developmental patterns. Understanding normative age curves for alcohol and tobacco use, as well as psychological distress, is essential for identifying deviations and co-occurring risks.

Despite differences in follow-up intervals and measurement tools, both cohorts showed a well-established peak in substance use during early adulthood. This pattern likely reflects the social and environmental changes (e.g., increased independence), psychological development (e.g., identity exploration) and ongoing brain development (e.g., decision-making) during this life phase (Degenhardt et al., 2016). The peak in daily cigarette smoking was particularly evident in the older TAM cohort. However, distinguishing between normative behavior and problematic use during this life stage can be challenging (Paus et al., 2008). Given that both cohorts are population-based, the observed age curves can be interpreted as reflecting normative development. Previous Finnish studies have shown that HED does not necessarily decline after early adulthood, especially among men, but may increase into the mid-forties, which was also evident in the TAM cohort (Härkönen & Mäkelä, 2011). This discrepancy compared to many other countries may be explained by Finland's traditionally drunkenness-oriented drinking culture, particularly among some men (Tigerstedt & Törrönen, 2007).

Age curves of distress were rather stable in the TAM cohort (with a small peak at age 32 years) and decreased in the FinnTwin16 cohort. Within each cohort, distress levels differed between sexes but followed similar trajectories. Prior research suggests that sex differences in distress persist into old age (Kuehner, 2017). However, developmental patterns of distress vary widely depending on measurement tools and age-related risk factors (Jorm, 2000). The increasing trend observed in TAM cohort aligns with typical findings in longitudinal studies. There were some within-study differences in the measurement of distress in the FinnTwin16 (age 16 vs. 25/35 years), which may explain the decreasing trend in this cohort. More studies are needed to disentangle age, period and cohort effects in psychological distress (Jorm, 2000). Trajectory analysis allows for a more nuanced understanding of these average curves, helping to distinguish between normative and non-normative development.

There is *heterogeneity in the age curves,* as shown in previous research, including earlier studies with these two cohorts (Berg et al., 2013; Jackson et al., 2009; Stephenson et al., 2024). The developmental perspective by Schulenberg et al. (2018) emphasizes that the development of HED, smoking behavior and psychological distress does not occur in isolation. This present study is among the few to address this perspective comprehensively by exploring all three developmental domains simultaneously, revealing three to five distinct joint patterns of HED, daily cigarette smoking, and distress. Stability in these trajectories was more common than fluctuation. In the most adverse development (Johnson et al., 2018; Mathers et al., 2006), a minority of individuals (e.g., 10% in TAM) were assigned to a trajectory group indicating persistently high levels of all three health concerns from adolescence into adulthood. Effective interventions to prevent the persistence of these problems should be targeted early in life. However, co-occurring mental health and substance use problems are often under-identified in adolescence. Due to stigma, adolescents may be more often diagnosed with psychiatric disorders rather than substance use disorder or both (Priester et al., 2016; Sterling et al., 2010). There is a need for developmentally appropriate treatment options for adolescents (Sterling et al., 2010).

The largest trajectory groups (61% in FinnTwin16; 30% in TAM) were characterized by consistently low levels of substance use and distress. To our knowledge, the only comparable study is that of Lee et al. (2018), which followed participants from age 14 years up to 29 years. The cohort studied by Lee et al. (2018) was similar to FinnTwin16 in terms of birth year and follow-up duration. The proportions of individuals in the “all low” (33%) and “all high” (12%) groups in their study were comparable to that in the TAM cohort. Substance use has generally been more prevalent in older cohorts (Helakorpi et al. 2004), particularly among men. While these similarities are notable, further research with additional cohorts is needed to clarify sources of variation, such as population structure, follow-up duration and attrition. Future studies should also extend follow-up into older adulthood because recent evidence suggests increasing substance use among older populations (Tigerstedt, Härkönen, et al., 2020), yet little is known about its co-occurrence with mental health problems. Overall, most trajectory groups showed little decrease in any of the three outcomes, especially in the older cohort with longer follow-up. Some groups did show a decline in substance use after a peak in early adulthood.

Two particularly important groups are those with high distress: one with low/moderate and one with high substance use. The identifying and distinguishing these stable patterns is valuable for mental health and substance use services. In this study, those with co-occurring problems were more often men, from non-nuclear families and with lower SEP background, consistent with previous literature (Lynch et al., 2021; Salom et al., 2014). Persistent depressive and anxiety symptoms require treatment, but substance use adds further complexity to clinical care (Baker et al., 2010). One clinical implication is the need to improve access to services and develop quality of treatment, a shift should be made towards a more client-centered approach to service provision (Priester et al., 2016). Evidence-based treatment for co-occurring problems should include a holistic bio-psychosocial approach, motivational strategies, services not limited by time nor abstinence, substance use counseling, multidisciplinary collaboration and outreach programs (Priester et al., 2016; Kola & Krusynski, 2010).

The sizes of the two trajectory groups varied between cohorts: “all high group” comprised 10% of participants in TAM and 26% in FinnTwin16, while the “high distress” group included 23% in TAM and 13% in FinnTwin16. These proportions may reflect clinically relevant subpopulations, but should be interpreted with caution, as latent class analysis involves balancing between the accuracy of representation of the trajectory groups in the real world and usability in analysis (Nagin & Odgers, 2010).

From a public health perspective, it is important that both alcohol and nicotine use are addressed in mental health and substance use services. However, alcohol often receives more attention (Baca & Yahne, 2009; Boatner et al., 2020; Hall & Prochaska, 2009). Previous research has shown that cigarette smoking dependence and smoking dependence motives related to heavy, automatic use and use to regulate affective states are associated with depression (Piirtola et al., 2021), highlighting the important independent role of daily smoking in these associations.

Two trajectory groups were identified only in the TAM cohort, which had a longer follow-up (36 vs. 19 years). Sensitivity analyses using only the first three time points in TAM revealed similar trajectory groups to those found when analyzing all five time points. The only trajectory group missing using a shorter follow-up time was “increased HED, low daily smoking and low distress”. This group, consisting mainly of men, reflecting the previously noted pattern in Finnish men's drinking behaviour, where HED does not decrease after early adulthood, but rather later in mid-adulthood (Berg et al. 2013; Härkönen & Mäkelä, 2011). It may be that this group would have been found also in the FinnTwin16 if the follow-up was longer. This highlights the importance of extending follow-up times beyond early adulthood when examining trajectories of substance use. This group should be better recognized in health services. In addition to prevention in adolescence, interventions should also target men whose drinking does not decline after early adulthood. However, also within-study differences in HED measures may play a role.

The other group found only in the TAM cohort was characterized by moderate levels of all indicators with a peak in daily cigarette smoking. Sensitivity analyses suggest this difference is not due to differences in the follow-up duration. Although the birth years of the cohorts are relatively close, cohort effects may still play a role because smoking was more prevalent in the older cohort, particularly among men (Helakorpi et al., 2004).

Another aspect of the developmental perspective concerns *long-term connections*, specifically the predictors and consequences of trajectory group membership. In line with our hypothesis, males and individuals with non-nuclear family and low parental SEP backgrounds were more likely to belong to groups with elevated substance use or distress compared to those with low levels across all outcomes. Men were more often assigned to substance use-oriented groups, while women were more often assigned to distress-oriented groups. These sex differences, evident in previous research, were confirmed here using modeling of all three outcomes.

Sensitivity analysis (data not shown) revealed similar trajectory groups for men and women, but these sex-stratified analyses also showed similar tendencies in sex differences. For example, in the TAM cohort, women in the high distress, low substance use group showed more pronounced distress than the overall group, while, in men, the increasing HED group was more pronounced than the distress group. This difference was also evident in the pooled analysis, supporting the decision to analyze combined data.

These sociodemographic risk factors, sex, family structure and SEP, have been previously linked to distress, cigarette smoking and HED individually. However, there is no clear consensus on whether these risks differ depending on whether individuals experience one, two or multiple co-occurring health concerns (or co-occurring fluctuation across the life span) (Salom et al., 2014; Green et al., 2012). The results of the present study do not point to an increased risk for co-occurrence because sex, compared to the reference group, was associated with all identified trajectory classes (except “all high” in the TAM) as was also other than nuclear family and manual socioeconomic background. The finding is consistent with the triple trajectory study by Lee et al. (2018). For example, in the TAM cohort, the magnitude of association regarding SEP was similar across all trajectory groups, suggesting that low socioeconomic status is a general risk factor for various adverse developmental paths. In addition to direct health risks, low SEP is associated with cultural attitudes and beliefs about mental health and substance use, as well as help-seeking, which form barriers for identification of mental health and substance use problems and their treatment (Priester et al., 2016). However, parental SEP was not associated with membership in the “increased HED” group in TAM or the “high distress, low substance use” group in FinnTwin16. These exceptions suggest that, although low SEP is a general risk factor, its influence may vary depending on the specific developmental pathway.

Another important component of the developmental perspective is the *broader sociocultural context*, which includes ecological levels ranging from macro-level societal structures to historical time. In this study design with only two partly overlapping macro-level contexts (Tampere area vs. national), it is difficult to analyze the role of macro-level factors in detail. However, some contextual factors should be considered. Both alcohol and tobacco are substances affecting health, but the development of their consumption in society has differed significantly in recent decades. HED has increased in Finland until the 2000s and then gradually started to decrease, with notable socioeconomic disparities (Tigerstedt, Mäkelä, et al., 2020). Tobacco use has generally decreased, except for snuff use (CAN, 2017). The cohorts were born 7–12 years apart. The older TAM cohort reached early adulthood during the economic boom of the late 1980s and experienced the severe recession of the 1990s. FinnTwin16 was in their twenties and in the middle of the transition from school to work in the mid-1990s during the recession. A Swedish study found cohort differences in alcohol use, but more so in total alcohol consumption than HED (Berg et al., 2020). A Finnish study of cohorts born between 1946 and 1977 found no cohort differences in alcohol use among men, but consistent increases in heavy drinking among women across successive cohorts, except for the youngest cohorts born after 1970, which resembled FinnTwin16 (Härkönen, 2013).

The TAM, although drawn from a single city, was representative of the national age cohort in terms of, marital status (Official Statistics Finland. Population structure, 2023a), and its educational level was comparable to urban population (Official Statistics Finland, Educational structure of population, 2023b).

Future research should aim to model the development of multiple health concerns simultaneously and identify both precursors and consequences of these heterogeneous trajectories. While the present study did not seek to establish causal paths, it is important to acknowledge that the associations are likely reciprocal and influenced by multiple underlying mechanisms. Previous studies have shown that associations between mental health and alcohol use persist even after controlling for confounding factors (Boden & Fergusson 2011; Savert & Hasin, 2016; Virtanen et al., 2020). Regarding smoking and mental health, some evidence suggests that shared genetics and familial liabilities may partly explain the association (Ranjit et al., 2019).

Many mental health problems begin in adolescence or early adulthood, and experimentation with alcohol or tobacco during this period may mark the onset of long-term substance use problems. Identifying individuals at risk for persistent problems is crucial, and places significant demands on substance use and mental health services. In clinical settings, it is important to consider not only current risk factors, but also the long-term developmental history of individuals.

## Methodological Considerations

The present study utilized data from two large prospective cohorts that followed participants across multiple life stages with relatively high participation rates. However, attrition was present in both cohorts. In the TAM cohort, male sex and poor school performance at age 16 years predicted a lower number of responses between ages 22 and 52 years (range 0–4 years), whereas substance use and distress at age 16 years did not (Berg et al., 2021). On average, participants responded to 3.6 out of five follow-ups. In FinnTwin16, male sex and low parental SEP at age 16 years predicted non-participation at age 35 years. It should also be noted that nearly all participants in the target populations of these cohorts were white, which may limit the generalizability of findings to more diverse populations.

Future research should aim to study substance use and psychological distress in greater detail. In this study, depressive and anxiety symptoms were combined into a general measure of psychological distress. Differentiating these domains in future studies may reveal somewhat distinct associations with substance use. Additionally, we combined non-daily smokers and non-smokers, and did not measure the amount of tobacco used.

While using multiple existing datasets offers clear advantages, retrospective harmonization poses challenges. Within-study differences in measurement across time points and between-study differences must be considered when interpreting results. For example, early assessments of HED were based on perceived drunkenness, whereas later assessments used more precise measures of alcohol quantity. Therefore, conclusions about the development of HED should be made cautiously, acknowledging these methodological shifts.

## Conclusions

The present study identified several distinct patterns in how HED, daily cigarette smoking and psychological distress co-occur and develop from adolescence to adulthood. Mostly, these joint patterns are stable and, although most individuals experience only mild issues with substance use and distress, a small subgroup consistently faces intertwined problems. These co-occurring concerns should be identified earlier, ideally already in adolescence, to enable timely and targeted support.

The trajectory groups identified in this study offer a new perspective on the complex interplay between substance use and mental health. Despite the high correlations between these domains, they are often addressed separately in the service sector (Morisano et al. 2014). Our findings underscore the need for a more holistic, person-centered approach that recognizes the heterogeneity in individuals’ developmental histories and tailors prevention and treatment accordingly. Life phase-specific interventions are needed to prevent the emergence and escalation of risky substance use and psychological distress, both as separate and co-occurring public health challenges.

## Appendix

[Fig fig4-14550725251408211] [Fig fig5-14550725251408211] [Fig fig6-14550725251408211] [Fig fig7-14550725251408211]
